# Brain Intraventricular Injection of Amyloid-β in Zebrafish Embryo Impairs Cognition and Increases Tau Phosphorylation, Effects Reversed by Lithium

**DOI:** 10.1371/journal.pone.0105862

**Published:** 2014-09-04

**Authors:** Laura Roesler Nery, Natalia Silva Eltz, Cristiana Hackman, Raphaela Fonseca, Stefani Altenhofen, Heydi Noriega Guerra, Vanessa Morais Freitas, Carla Denise Bonan, Monica Ryff Moreira Roca Vianna

**Affiliations:** 1 ZebLab & Laboratório de Biologia e Desenvolvimento do Sistema Nervoso, Faculdade de Biociências, Pontifícia Universidade Católica do Rio Grande do Sul, Porto Alegre, Rio Grande do Sul, Brazil; 2 ZebLab & Laboratório de Neuroquímica e Psicofarmacologia, Faculdade de Biociências, Pontifícia Universidade Católica do Rio Grande do Sul, Porto Alegre, Rio Grande do Sul, Brazil; 3 Department of Cell and Developmental Biology, Institute of Biomedical Sciences, University of Sao Paulo, Sao Paulo, Brazil; Federal University of Rio de Janeiro, Brazil

## Abstract

Alzheimer’s disease (AD) is a devastating neurodegenerative disorder with no effective treatment and commonly diagnosed only on late stages. Amyloid-β (Aβ) accumulation and exacerbated tau phosphorylation are molecular hallmarks of AD implicated in cognitive deficits and synaptic and neuronal loss. The Aβ and tau connection is beginning to be elucidated and attributed to interaction with different components of common signaling pathways. Recent evidences suggest that non-fibrillary Aβ forms bind to membrane receptors and modulate GSK-3β activity, which in turn phosphorylates the microtubule-associated tau protein leading to axonal disruption and toxic accumulation. Available AD animal models, ranging from rodent to invertebrates, significantly contributed to our current knowledge, but complementary platforms for mechanistic and candidate drug screenings remain critical for the identification of early stage biomarkers and potential disease-modifying therapies. Here we show that Aβ1–42 injection in the hindbrain ventricle of 24 hpf zebrafish embryos results in specific cognitive deficits and increased tau phosphorylation in GSK-3β target residues at 5dpf larvae. These effects are reversed by lithium incubation and not accompanied by apoptotic markers. We believe this may represent a straightforward platform useful to identification of cellular and molecular mechanisms of early stage AD-like symptoms and the effects of neuroactive molecules in pharmacological screenings.

## Introduction

Alzheimer’s disease (AD) is the most prevalent cause of dementia currently affecting 30 million individuals and expected to quadruplicate until 2050 [1]. AD symptoms include progressive cognitive decline due to synaptic and neuronal deterioration and the lack of effective treatments results in extensive care with elevated costs for several years [1], inexorably culminating in death. Despite abundant information about altered cellular events in sporadic AD, its effective treatment depends on understanding sequential disease mechanisms in order to identify potential treatment-targets in early stages.

AD is characterized by deposition and aggregation of Amyloid-β (Aβ) protein on the extracellular space [1] [2] while the microtubule-associated tau protein becomes abnormally phosphorylated, disrupts cytoskeletal organization and accumulates on toxic neurofibrillary tangles in the cytosol [3].

Recent studies support new features of the non-fibrillary and soluble Aβ peptide forms before aggregation that are believed to be prevalent in initial disease stages [4] [5] [6]. A correlation between soluble Aβ and increased tau protein phosphorylation has been demonstrated [7] [8] [9] [10]. Tau phosphorylation (tau-p) can be reversed by pharmacological inhibition of specific tau-kinases such as Glycogen synthase kinase-3β (GSK-3β) in rodents [11] [12] and AD patients [13]. The missing link between Aβ and tau accumulation could be the Aβ soluble peptide forms that bind to membrane receptors and modulate GSK-3β activity [6] [7].

A better understanding of AD progression and its cellular and molecular mechanisms depends on animal models that mimic specific disease aspects. Intracerebral infusion of Aβ protein in rodents has contributed significantly to understanding the AD underlying machinery [14] [15] [16]. Complementary models to study molecular aspects of AD include the fruit fly drosophila [17] and the nematode *C. elegans* [18]. However, the need of complementary platforms for mechanistic and candidate drug screenings remains. Zebrafish has emerged in the last decade as an advantageous model organism for high-throughput pharmacological screenings of neuroactive compounds [19] [20] [21] [22] and recently shown to share 84% of the genes known to be associated with human diseases [23], including those related to AD [20].

In this context, we propose a complementary model to further dissect the pathological effects of soluble Aβ and simultaneously screen for potential neuroprotective molecules. Our model is based on the hindbrain ventricle injection of the Aβ1-42 peptide in 24 hpf zebrafish embryos and results in specific behavioral and molecular effects that resemble early stage AD features. Specific cognitive deficits and tau-phosphorylation in residues associated to early AD stages were reversed by lithium chloride, a GSK-3β inhibitor.

## Materials and Methods

### Animals

Adult wild type zebrafish were kept and bred according to standard procedures in an automated re-circulating system (Tecniplast) at a density of 1.5 fish per liter with a constant light-dark cycle (14–10 h) [24]. For breeding, females and male (1∶2) placed in breeding tanks (Tecniplast) overnight were separated by a transparent barrier that was removed after lights went on in the following morning. Embryos were collected after 15 min and transferred to sterile 6-well cell culture plates (20 embryos per well) kept in incubators at 28.5°C and controlled 14∶10 hours light-dark cycle. Embryos and larvae had their mortality and general morphology daily monitored under an inverted stereomicroscope (Nikon).

### Ethics Statement

All protocols were approved by the Institutional Animal Care Committee (CEUA-PUCRS, permit number 0107/12), followed the Brazilian legislation (no.11.794/08) and conducted according to the Canadian Council on Animal Care guidelines for the use of fish in research [25].

### Treatment and Brain Ventricle Injection

**Figure 1:**
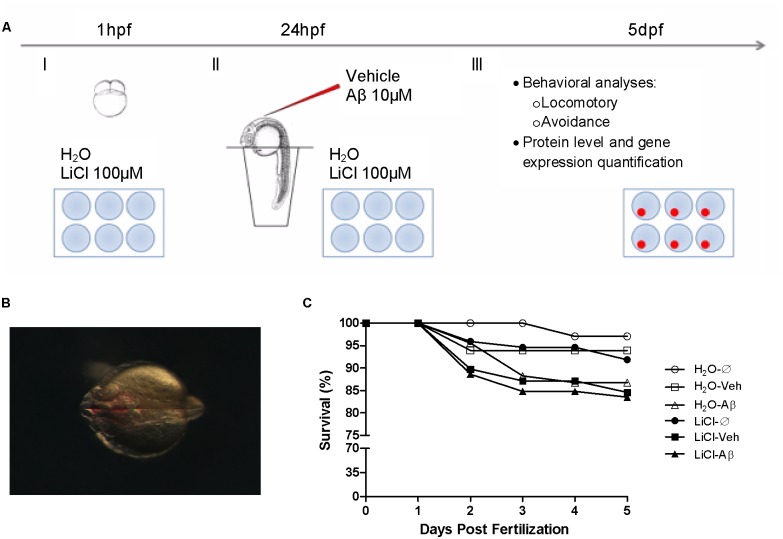
Experimental design and Aβ intraventricular injection effect on survival. A, Experimental procedures time-line, (I) at 1 hpf embryos were placed in 6-well plates and exposed to LiCl 100 µM or H_2_O; (II) at 24 hpf embryos were removed from their chorion, and injected with Aβ1-42 10 µM or its vehicle; LiCl and H_2_O solutions were replaced daily throughout the experiment; (III) at 5dpf larvae behavior were evaluated and samples for protein and gene expression quantification were obtained. B, Representative image of Aβ (red) injected on the brain ventricle area. C, Kaplan-Meier survival comparison for all groups throughout the experiment showed significant effects (Log-rank (Mantel-Cox) test, p = 0.0415, N = 60 in triplicates) that were not statistically significant when individual comparisons were performed.

Embryos were treated with system water (RO water equilibrated with Instant Ocean salts - H_2_O) or 100 µM Lithium Chloride (LiCl) (Synth) [26] diluted in system water from 1 hour post-fertilization (hpf) until 5 days post-fertilization (dpf) (Figure 1A). Medium was daily replaced and controlled for pH.

At 24 hpf all embryos had their chorion removed for the brain ventricle injection procedure according to Gutzman and Sive [27]. Embryos were anesthetized with Tricaine (Sigma Aldrich) and placed in wells on 0.75%-agar coated dishes under the stereomicroscope so that the brain ventricle was visible and the embryo unmoving (Figure 1A). The microinjection was performed using a micromanipulator (Narishige) attached to a Picoliter injection pump (Warner Instruments). The injection needle was placed on the roof plate of the hindbrain and 5–10 nl of a 10 µM Aβ1-42 in 1% DMSO 0.5% Phenol Red Phosphate Buffered Saline (PBS) solution (Aβ) was injected while control group animals received the equivalent 1% DMSO 0.5% Phenol Red in PBS vehicle (veh). In less than 10 minutes each animal was returned to the incubator. In addition to the resulting H_2_O-veh, LiCl-veh, H_2_O_–_Aβ, LiCl-Aβ, additional control groups of uninjected animals treated with water (H_2_O_–_Ø) or LiCl (LiCl-Ø) composed final 6 experimental groups.

The Aβ1-42 peptide (Sigma Aldrich) was prepared following manufacturer instructions in DMSO 100% to a final concentration of 1000 µM and diluted to the final 10 µM according to Cunvong et al. [28]. To prevent Aβ aggregation into the fibrillary form, the solution was maintained at a maximum temperature of 28°C and the pH was adjusted to 7.0. Diluted Aβ1-42 was “ran-true” in SDS-PAGE gel and a band of an approximately molecular weight of 40 kDa was observed [10].

### Locomotor behavior

5dpf larvae from all experimental groups (N = 10 in triplicates) were individually placed in a 24-well plate filled with 3 ml of system water for locomotory performance analysis during a 5-min session following 1-min acclimation. The performance was video recorded using a digital HD webcam (Logitech) for automated analysis (ANYmaze, Stoelting). Total distance travelled, mean speed, time mobile and absolute turn angle were considered the main parameters of exploration of a new environment.

### Bouncing-Ball Avoidance behavior

After the exploratory evaluation, larvae were placed in 6-well plate (5 larvae per well, N = 10 in triplicates) over a LCD monitor for cognitive ability and avoidance responses to a visual stimulus (a 1.35 cm diameter red bouncing ball) in a protocol adapted from Pelkowski et al. [29] during a 5-min session following 2-min acclimation. The red bouncing ball travelled from left to right over a straight 2 cm trajectory on half of the well area (stimuli area) (Figure 1A) which animals avoided by swimming to the other non-stimuli half of the well. The number of larvae on the non-stimuli area during the 5-min session was considered indicative of their cognitive ability.

### Western Blot

Euthanized 5dpf larvae (Figure 1A) had their encephalon dissected (pool of 20 animals, N = 3 in triplicates) and stored at −80°C in protease inhibitor cocktail (Sigma Aldrich) until homogenization with RIPA (Sigma Aldrich) and protein separation on 12% SDS-polyacrylamide gel with sample buffer (0.025% BPB). Proteins were transferred to a nitrocellulose membrane and blocked with 5% bovine serum albumin on TBST. Primary Antibodies were diluted on the blocking solution at the following concentrations: Rb-β-actin (Anaspec; 1∶1000); Ms-Phospho-PHF-tau pSer202/Thr205 AT8 (Pierce; 1∶500); Rb-p53 (Anaspec; 1∶1000); Rb-bax (Anaspec; 1∶750) and Rb-caspase-8 (Anaspec; 1∶750); and incubated overnight, washed three time with TBST and incubated for 1 hour with secondary antibody diluted in 5% Albumin in TBST at the concentrations of Goat-anti-Rabbit IgG (Sigma Aldrich; 1∶2000) and Goat-anti-Mouse IgG (Abcam; 1∶2000). Membranes were washed with TBST, incubated with ECL (Abcam) and scanned for further densitometric quantification of replicated gels using the software Carestream. After exposure, membranes were washed in TBST to remove ECL solution and antibodies were striped out by dehybridization with (2% SDS, 50 mM Tris pH 6.8 and 100 mM β-mercaptoethanol), and incubated with other antibodies using the same actin control. Total protein levels were normalized according to each sample’s β-actin levels.

### Real time PCR

RNA isolation and cDNA synthesis were performed according to the manufacturer’s instruction. Briefly, 5dpf larvae had their encephalon dissected (Figure 1A) (pool of 20 animals, N = 6 in duplicates) placed in TRIzol (Invitrogen), frozen in liquid nitrogen and maintained at −80°C. mRNA was isolated and cDNA were synthesized with SuperScriptIII First-Strand Synthesis SuperMix (Invitrogen).

For all genes, qRT-PCRs were performed using SYBR green dye [30]. Standard reactions were performed with a total 25 µL per well, on an Applied Biosystems 7500 real-time PCR system, and the primer final concentration were 0.1 µM. The primers sequences were described previously by Tang [30] for constitutive genes: b-actin1 F:5′-CGAGCTGTCTTCCCATCCA-3′, R:5′-TCACC-AACGTZGCTGTCTTTCTG-3′; ef1a F:5′-CTGGAGGCCAGCTCAAACAT–3′, R:5′-ATCAAGAAGAGTAGTACCGCTAGCATTAC-3′; and rpl13a F:5′-TCT-GGAGGACTGTAAGAGGTATGC-3′, R:5′-AGACGCACAATCTTGAGAGCAG-3′. The target genes were previously described by Jung et al. [31]: p53 F:5′-CTATAAGAAGTCCGAGCATGTGG-3′, R:5′-GGTTTTGGTCTCTTGGTCTTCT-3′; bax-a F:5′-GAGCTGCACTTCTCAACAACTTT-3′, R:5′-CTGGTTGAAATAG-CCTTGATGAC-3′ and bcl-2 F:5′-TTGTGGAGAAATACCTCAAGCAT-3′, R:5′-GAGTCTCTCTGCTGACCGTACAT-3′. Amplification and dissociation curves generated by the software were used for gene expression analysis.

Threshold Cycle (Ct) values were obtained for each gene. Following exclusion of non-amplificating samples, raw fluorescence data was exported to the software LinRegPCR 12.x to determine the PCR amplification efficiency of each sample. PCR efficiency of each sample, together with Ct values, was used to calculate a relative gene expression value for each transcript according to Pfaffi [32].

### Statistical analyzes

Survival throughout the 5 experimental days was analyzed by Kaplan-Meier test. Data from all other approaches was parametrically analyzed using two-way ANOVA (using treatment and injection as factors) followed by Bonferroni post-hoc test. Student-t test was used to evaluate specific data when needed. The level of significance was considered p<0.05.

## Results

First, we adapted Gutzman and Sive [27] protocol for 24 hpf zebrafish brain ventricle injection, and in addition to a dye tracer to ensure correct injection positioning (Figure 1B), we injected animals with 10 µM of soluble Aβ1-42 in 1% DMSO (Aβ-injected group) or only 1% DMSO vehicle (veh-injected group) (Figure 1). To our knowledge, this is the first demonstration of ventricular brain Aβ injection in zebrafish in addition to the dye used for validation. No morphological alteration or injection effect was observed in any of the evaluated parameters throughout the experiments. In addition to those injection groups, we also controlled the effects of the microinjection puncture and did not find any deleterious effect on survival, deformities or behavior (data not shown).

Survival rates were analyzed by Kaplan-Meier test and results indicated a significant difference on survival rate when all groups were compared (Log-rank (Mantel-Cox) test p = 0.0415, N = 60). The decrease in survival on H_2_O_–_Aβ (86.7%), LiCl-veh (84.5%) and LiCl-Aβ (83.5%) groups was not significant when individual comparisons were performed (Figure 1C). This effect is discrete and challenging to interpret since it was not homogeneously distributed among treatment or injection factors. It may be associated to the high and uniform within group survival rates from most groups (mean survival of H_2_O-treated non-injected individuals was 97.06%) in comparison to commonly observed values of around 75% in these developmental stages [33].

**Figure 2:**
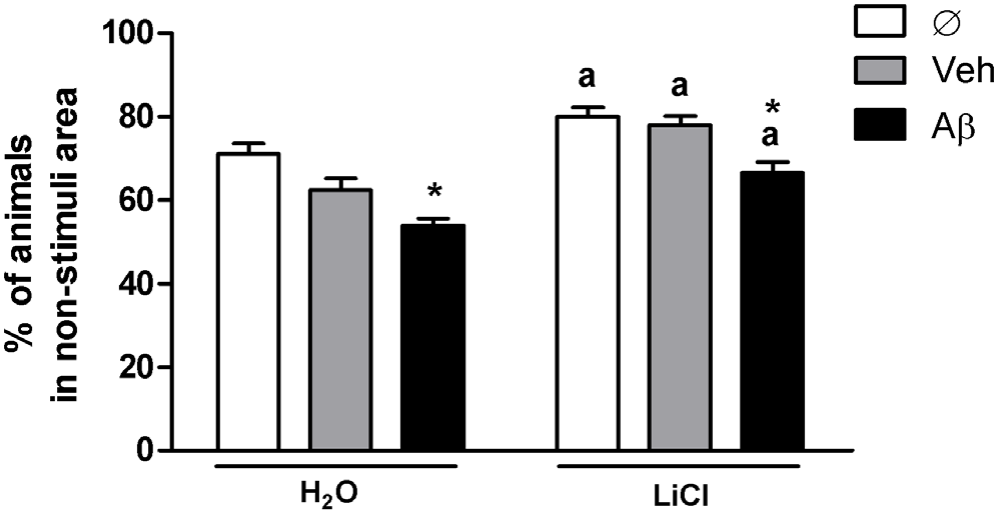
Intraventricular Aβ injection significantly impairs avoidance of an aversive stimulus. 5dpf larvae escape behavior from an aversive stimulus (charts were plotted with means and SD escape responses to a non-stimuli area). Two-way ANOVA followed by Bonferroni demonstrated a significant effect of treatment factor (H_2_O and LiCl) (p<0.0001; F_(1,166)_ = 40.77; N = 10 in triplicates). Aβ injected animals showed diminished escape responses when compared to their vehicle control group in H_2_O and LiCl-treated groups (* indicates p<0.05 for both comparisons). LiCl treatment increased escape responses in all groups when compared to their respective H_2_O-treated equivalent (a indicates p<0.05 for noninjected Ø groups; p<0.0001 for veh-injected groups and p<0.001 for Aβ-injected groups in Student-t test.

Considering that AD behavioral symptoms in early stages include only mild cognitive deficits, which are several times misdiagnosed and confounded with normal aging even thought not accompanied by aging-characteristic motor deficits [6], we evaluated locomotion separately from cognitive behavior in 5dpf larva. Individually locomotor evaluation and exploratory parameters showed no statistical differences between groups, including total distance travelled (p = 0.250 F_(1,179)_ = 1.40), mean speed (p = 0.181 F_(1,185)_ = 1.72), time spent mobile (p = 0.70 F_(1,182)_ = 0.36) and path absolute turn angle, a motor coordination parameter [34], (p = 0.409 F_(1,186)_ = 0.90) using two-way ANOVA (data not shown). However, when cognitive escaping responses from an aversive stimulus was evaluated, we observed a significant effect of Aβ-injection and LiCl-treatment (two-way ANOVA p<0.0001 F_(1,166)_ = 40.77 on treatment effect, and p<0.0001 F_(2,166)_ = 21.13 on injection effect) (Figure 2). Bonferroni posttest showed that Aβ-injected animals avoided the aversive stimulus less effectively than animals injected with vehicle on both treatments (p<0.05). When comparing only H_2_O_–_Aβ and LiCl-Aβ animals, LiCl significantly improved the scape response to the stimuli (p = 0.0002, Student-t test), suggesting protective effect from Aβ-induced cognitive deficits. Additionally, the lithium beneficial effect on cognition was observed in all groups when compared to their respective water-treated controls.

**Figure 3:**
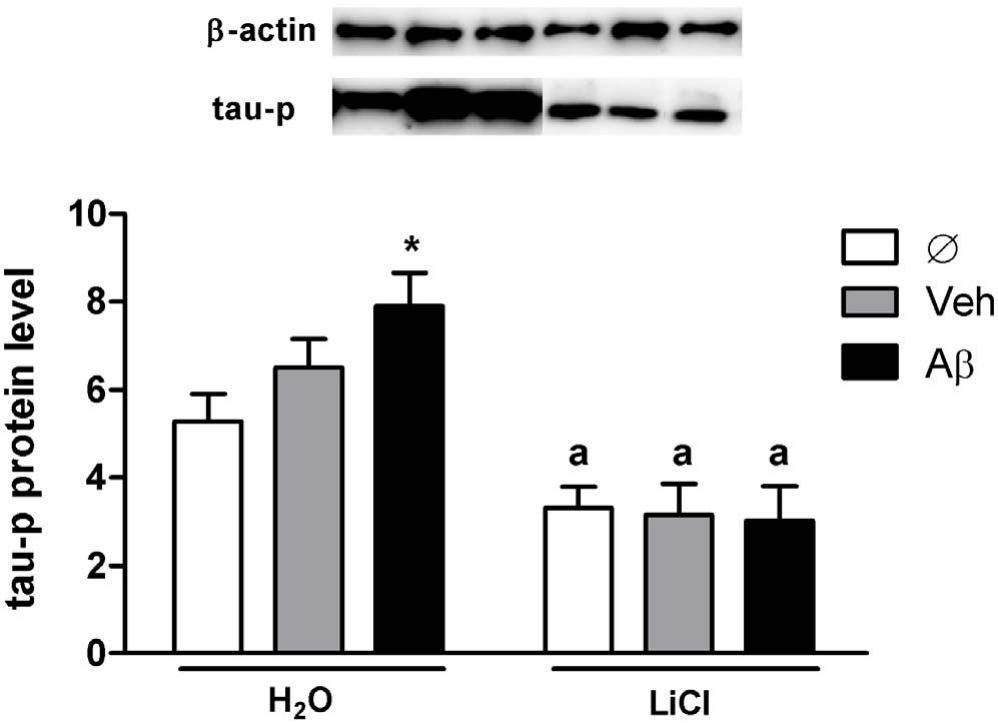
Intraventricular Aβ injection increases tau-p at Ser202 and Thr205 residues and this effect is reversed by lithium treatment. Representative Western blots showing immunoreactivity to phosphorylated tau protein normalized to β-actin and quantification of absorbance (charts were plotted with means and SD). Two-way ANOVA followed by Bonferroni demonstrated a significant effect of treatment factor (p<0.0001, F_(1,42)_ = 296.02; N = 3 in triplicates). H_2_O_–_Aβ injected animals showed increased levels of tau phosphorylation in relation to H_2_O-veh (*p<0.001). LiCl treatment decreased tau-p in all groups when compared to their respective H_2_O-treated equivalent (a indicates p<0.0001 in Student-t test for all comparisons).

To investigate if the selective cognitive deficits induced by Aβ injection and prevented by LiCl were accompanied by tau phosphorylation, we measured the level of Ser202 and Thr205 phosphorylation (Figure 3). These residues are GSK-3β targets [11] [35] [36] known to be increased in aged individuals and early-stage AD patients [6]. Our results showed again significant effects of Aβ injection and a general protective effect of the GSK-3β inhibitor LiCl (Two-way ANOVA p<0.0001; F _(1,42)_ = 296,00 on treatment effect, and p<0.0001 F_(2,42)_ = 11.77 on injection effect) (Figure 3). Untreated Aβ-injected animals (H_2_O_–_Aβ) displayed significantly higher levels of tau protein phosphorylation (p<0.001, Bonferroni posttest) when compared to H_2_O-veh. H_2_O-veh animals also displayed increased p-tau in relation to non-injected animals (H_2_O_–_Ø) (p<0.01, Bonferroni posttest), which may be attributed to DMSO toxicity even at very low concentrations. Interestingly, when LiCl-treated groups were paired with their respective H_2_O-treated equivalent groups, all comparisons showed a lithium significant effect decreasing tau-p levels (p<0.0001 for all groups; Student-t tests), which was more prominent between H_2_O_–_Aβ and LiCl-Aβ, in resemblance to the cognitive data (Figure 2). These protective effects of LiCl over the basal and Aβ-induced phosphorylation have been previously shown in other systems [37] [38] [39] but not in zebrafish.

**Figure 4:**
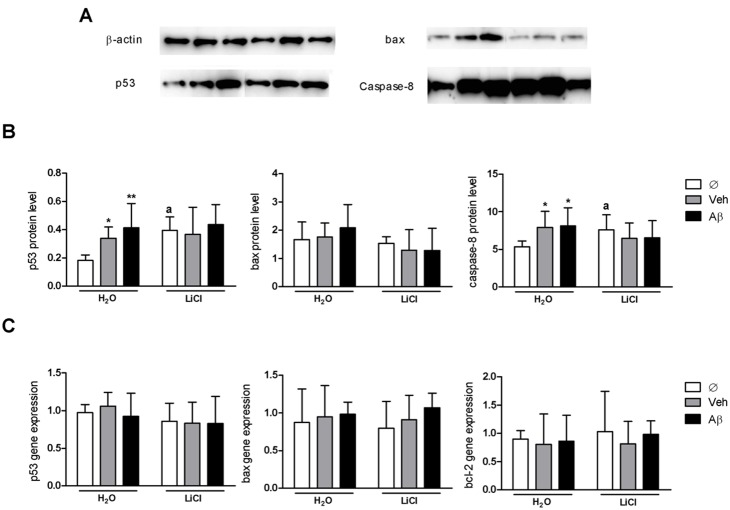
Intraventricular injection alters apoptotic targets. A, representative Western blots showing immunoreactivity of indicated proteins normalized to β-actin. B, Western blots quantification of absorbance (charts were plotted with means and SD). Two-way ANOVA followed by Bonferroni posttest didn’t show significant differences (p = 0.1153, F_(2,41)_ = 2.28 for p53; p = 0.3063, F_(2,48)_ = 1.21 for bax; p = 0.4420, F_(2,45)_ = 0.83 for caspase-8; N = 3 in triplicates) in Aβ injected animals compared to their vehicle control group in H_2_O or LiCl-treated groups. P53 and caspase-8 levels differed between H_2_O-veh and H_2_O_–_Aβ and noninjected H_2_O_–_Ø controls (*p<0.05, **p<0.01). Among noninjected animals, LiCl treatment increased p53 and caspase-8 protein levels compared to their respective H_2_O-treated equivalent (a indicates p<0.01 for caspase-8 and p<0.0001 for p53 in Student-t test). C, q-PCR analysis normalized to three constitutive genes (b-actin, rpl13a and ef1a) (charts were plotted with means and SD). Two-way ANOVA followed by Bonferroni posttest didn’t show significant differences on gene expression (p = 0.5473, F_(2,88)_ = 0.61 for p53; p = 0.7313, F_(2,48)_ = 0.31 for bax; p = 0.8822, F_(2,50)_ = 0.13 for bcl-2; N = 6 in duplicates).

Tau abnormal phosphorylation has been associated to AD progression, axonal disruption, synaptic loss and neuronal death (reviewed in [6]). Aβ-induced effects on cognition (Figure 2) and tau-phosphorylation (Figure 3) suggested that our model resembles early AD stages that were not related with cell death. We quantified protein and transcription levels of apoptosis-associated proteins that were previously suggested to be associated to Aβ-toxicity and neurodegeneration including p53, caspase-8, bax-a and non-apoptotic marker such as bcl-2 (Figure 4). Importantly, Aβ-injected (H_2_O_–_Aβ) animals did not differ from their respective vehicle-injected controls (H_2_O-veh) or from LiCl-Aβ animals in any comparison, suggesting no specific Aβ-induced effect on apoptosis and associated LiCl neuroprotective effect. No alterations on bax (p = 0.3063; F_(2,48)_ = 1.21 Two-way ANOVA) protein level were observed despite of significant effects on p53 (p = 0.035; F_(1,41)_ = 4.758 Two-way ANOVA) and caspase-8 (p = 0,0114; F_(1,48)_ = 6,916 Two-way ANOVA) protein levels (Figure 4B) due to both H_2_O_–_Aβ and H_2_O-veh higher protein levels regarding H_2_O_–_Ø animals (p<0.01 and p<0.05 respectively; Bonferroni posttest). These effects likely result from DMSO toxic effects [40]. Surprisingly, non-injected LiCl-treated animals showed higher levels of these same proteins when compared to their H_2_O_–_Ø controls (p<0.01 for both comparisons, Student-t tests). Messenger RNA levels of p53, bax and bcl-2 were not altered (p = 0.5473 F_(1,88)_ = 0.6069; p = 0.7313 F_(1,48)_ = 0.3149; p = 0.8822 F_(1,50)_ = 0.1257 respectively) (Figure 4C). These results suggest a scenario with undetectable alterations on apoptotic-associated proteins that may also resemble early stages of AD.

## Discussion

Early AD stages, usually misdiagnosed and confounded with aging associated symptoms, are characterized by subtle cognitive deficits, rising brain Aβ levels that progressively diffuse and oligomerize until aggregation in fibrils, and tau altered phosphorylation [1] [6] [41]. The absence of robust biomarkers for early diagnosis of AD is a major impediment for cost-effective and successful clinical trials of potential agents that could significantly slow or even prevent disease progression.

We established a straightforward low-cost platform for both biomarker identification and high-throughput preclinical pharmacological screenings in a prominent model organism. In the last decade zebrafish has emerged as a powerful model for drug screenings, genetic studies and disease modeling [22] [42]. In addition to the significant genetic similarity to humans, homologue genes encoding several proteins related to AD were identified [20] [21] [42], including the amyloid precursor protein (APP) with 80% of conservation [20]. This is the first report of Aβ brain injection in zebrafish, in parallel to Cunvong et al. [28] that injected the same peptide in the retina and Cameron et al. [43] that exposed embryos to amyloid-β 1–42 in the water. The original brain ventricle microinjection protocol from Gutzman and Sive [27] for 24 hpf zebrafish embryos was adapted and proved feasible in large scale, requiring a relatively accessible setup in which trained experimenters injected one embryo every 10 minutes, with no impact on animals survival.

Our behavioral results showed specific cognitive deficits in animals injected with Aβ peptide, which corroborates with findings in rodent that correlate Aβ peptide accumulation and memory impairment [44] [45] [46]. Several studies correlate Aβ-induced cognitive impairments with GSK-3β phosphorylation of tau protein residues resulting in cytoskeletal disorganization, synaptic loss and axonal disruption [8] [10]. We quantified the amount of early-phosphorylated tau protein and observed an increase on GSK-3β target residues Ser202 and Thr205 on animals injected with Aβ that was reversed by continuous 5dpf lithium exposure. Interestingly, LiCl had positive effects *per se* on cognition and tau basal phosphorylation that support its traditional view as a neuroprotective agent in low doses, with beneficial effects on memory [47] [48], long-term potentiation (LTP) [49] and tau phosphorylation [12] [50]. Those effects may be a result of basal GSK-3β activity inhibition, as suggested by Noble et al. [50] when observing reduced markers of tauopathy in transgenic mice after lithium. Maguschak and Ressler [51] also used mice to demonstrate a lithium-induced β-catenin increase associated to improved memory formation.

Magdesian and collaborators [9] reported that Aβ binds to Wnt receptor Frizzled inhibiting the Wnt canonical signaling pathway and therefore permitting GSK-3β activity. In corroboration with other studies [10] [11] [50] our results suggest that Aβ is associated with tau-increased phosphorylation by GSK-3β, reinforcing the therapeutic potential of GSK-3β inhibitors [12].

Compatible with an early AD scenario, we did not observe any specific effect of Aβ over protein and gene expression levels associated with apoptosis when Aβ-injected group was compared to its vehicle-injected controls. We found, however, an increase on p53 and caspase-8 protein levels on both injected groups (vehicle and Aβ) when compared to non-injected animals, which could be attributed to vehicle components [52] [53] [54]. Hanslick et al. [40] showed that DMSO has an apoptotic effect on mice developing central nervous system exposed to LiCl during the postnatal developmental stages. Surprisingly, our data also showed that lithium treated animals also have increased p53 and caspase-8 when compared to untreated non-injected animals. It is known that both apoptosis [55] and tau-phosphorylation in those residues [56] occur naturally during development and aging. It is also known that LiCl treatment can play different roles depending on cell type, system and developmental stage [57] [58] [59] [60]. In a developmental changing scenario, where neuron pathways are being formed and connection refinement is necessary [55], opposing effects on apoptotic pathways may be observed. In summary, we showed that Aβ1-42 injection in the ventricular region of 24 hpf zebrafish embryos induce cognitive deficits and an increase in tau phosphorylation, which were reversed by lithium incubation. We were not able to identify apoptosis and neurofibrillary tangles in animals submitted to this procedure at 5dpf and subsequent studies using this system should aim to characterize other cellular processes and molecular targets. We believe our model may represent a straightforward platform useful to identify mechanisms resembling early stage AD and the effect of neuroactive molecules in pharmacological screenings.
